# Perception of Neighborhood Characteristics During Childhood and Cognitive Health Among Older Adults in China

**DOI:** 10.1093/geroni/igaa057.1419

**Published:** 2020-12-16

**Authors:** Changmin Peng, Sae Hwang Han, Jeffrey Burr

**Affiliations:** 1 University of Massachusetts Boston, Boston, Massachusetts, United States; 2 University of Texas at Austin, Austin, Texas, United States

## Abstract

Neighborhood environments shape the availability of resources for social engagement and social interaction, which are associated with better health outcomes. However, these contextual factors are also considered sources of potential social distress and tension, increasing the risk of subsequent health deficits, including cognitive decline. Our understanding of the linkage between childhood neighborhood environments and cognitive functioning in later life is limited. This study employed three waves of nationally representative data from the China Health and Retirement Longitudinal Study (2011-2015; N = 11,105) to investigate the relationship between self-reported neighborhood social cohesion during childhood (i.e., neighborhood safety, neighbors willing to help, and close-knit neighborhood) and cognitive functioning (Chinese version of TICS). We employed latent growth curve modeling to test hypotheses relating to life course models of childhood conditions and later life cognitive functioning (the long arm of childhood). The results showed that perceptions regarding the willingness of neighbors to help and close-knit neighborhood characteristics during childhood were positively associated with levels of later life cognitive function. Further, growing up in a neighborhood characterized by the willingness of neighbors to help others was negatively associated with the rate of cognitive decline, net of childhood and adulthood covariates. Self-report of neighborhood safety during childhood was unrelated to cognitive function (level and change). These findings underscored the long-term ramifications of childhood conditions as potential risk factors for later-life cognitive health. Social cohesion at the neighborhood level as experienced during childhood may be a protective factor for healthy cognitive aging among older Chinese adults.

